# Association Between Bipolar Disorder and Low Bone Mass: A Cross-Sectional Study With Newly Diagnosed, Drug-Naïve Patients

**DOI:** 10.3389/fpsyt.2020.00530

**Published:** 2020-06-10

**Authors:** Sujuan Li, Yan Qui, Ziwei Teng, Jindong Chen, Dongyu Kang, Hui Tang, Hui Xiang, Chujun Wu, Yuxi Tan, Lu Wang, Yanyi Yang, Bolun Wang, Haishan Wu

**Affiliations:** ^1^Department of Psychiatry, The Second Xiangya Hospital of Central South University, Changsha, China; ^2^National Clinical Research Center for Mental Disorders, Changsha, China; ^3^Health Management Center, The Second Xiangya Hospital of Central South University, Changsha, China; ^4^Department of Orthopedics, The Second Xiangya Hospital of Central South University, Changsha, China

**Keywords:** bone mineral density (BMD), drug-naïve, bipolar disorder, body mass index (BMI), gender difference

## Abstract

**Background:**

Medical comorbidities in people with mental disorders have recently gained more attention. People with bipolar disorder (BD) often have comorbid low bone mass, which is associated with increased fracture risk and related severe outcomes. However, few clinical studies on bone metabolism in BD patients are available. This study was designed to assess bone mineral density (BMD) and related influencing factors in a sample of newly diagnosed, drug-naïve individuals with BD and age- and sex-matched healthy controls.

**Methods:**

Sixty-one drug-naïve individuals with BD (DSM-V) and 95 healthy volunteers had their lumbar spine (L1–L4) and left hip (Neck/Troch/Ward's) BMD determined by dual-energy X-ray absorptiometry. Besides, sociodemographic and clinical assessment were collected. Between-group comparisons and within subgroup analysis were performed.

**Results:**

Drug-naïve patients with BD had significantly lower BMD in comparison to healthy controls in multiple sites (L1, L3, Neck, Troch, Ward's, and total hip). On subgroup analysis, overweight individuals with BD had higher bone mass, while females presented reduced BMD. Binary logistic regression showed that low BMD in multiple regions was associated with BD diagnosis, body mass index (BMI), gender, and age.

**Conclusion:**

Drug-naïve individuals with BD have lower BMD when compared to an age- and gender-matched healthy control sample. Low BMI and female gender are factors associated with this outcome. The underlying pathological mechanisms of BD comorbid with osteoporosis should be further explored.

**Clinical Trial Registration:**

www.chictr.org.cn, identifier ChiCTR190002137.

## Introduction

Bipolar disorder (BD) is a chronic mental illness characterized by recurrent manic (or hypomanic) and depressive episodes that manifest throughout the lifespan, usually starting during adolescence or early adulthood (1). It is a highly incident and frequent condition, with estimated lifetime prevalence of 0.81–1.29% and no gender, race, or ethnic predilection (2). Although typical mood shifts during BD exacerbations can negatively influence individual functionality, significant impairment may also take place in-between depressive or manic episodes (3). During these periods, comorbid medical conditions additionally lead to declined quality of life, prolonged and complex treatment cycles, and higher mortality. The later may be either a consequence of increased suicide risk or severe outcomes following prior medical conditions, such as stroke and myocardial infarction, for instance (4).

Bone mineral density (BMD) reduction, with osteoporosis as the most severe form, is a prevalent systemic disease with multiple causes. This disorder is characterized by an imbalance between bone resorption and bone formation with consequent low BMD and skeletal microarchitecture deterioration (5). The resulting inflation of bone frailty leads to an increase in fracture risk (6). Around one-third of fall-related deaths are attributable to fractures due to low BMD. In addition to the increased mortality, fractures may result in permanent disability (7). Accordingly, patients with osteoporosis lose an average of 6.1 disability-adjusted life years, and this effect increases in severity if osteoporosis is comorbid with BD (8, 9). Osteoporosis is one of the most common medical comorbidities of people with BD, next to obesity and cardiovascular diseases. The incidence of osteoporosis in this population is around 7%, two times higher than that in the general population (10).

The mechanisms of bone loss in individuals with BD are complex and still controversial, with therapeutic drugs mainly implicated. Bolton and colleagues, for instance, showed that the use of psychotropic medications by postmenopausal women with multiple mental disorders, including BD, is associated with an increased risk for low BMD regardless of the specific diagnosis (11). The medical management of patients with BD is complex (12), with drug regimens that usually contain antidepressants, antipsychotics, and mood stabilizers, all of which may affect BMD (13, 14). However, recent studies set more focus on the potential overlap of pathological mechanisms between osteoporosis or low bone mass and BD, for instance oxidative stress (15) and inflammation pathways (16). Therefore, the mere diagnosis of BD itself, regardless of intake of psychotropic drugs and unhealthy lifestyles, may be an independent risk factor for low BMD. In keeping with this hypothesis, we designed a cross-sectional study to objectively assess the BMD of patients with a newly diagnosis of BD prior to the initiation of any psychotropic drug and to compare measurements of this outcome to those of healthy controls. We further aimed to explore possible variables that could be associated with low BMD in this sample.

## Methods

### Participants

From March 2019 to July 2019, individuals with BD were consecutively recruited from the psychiatry clinic of the Second Xiangya Hospital of Central South University, Changsha, China. The inclusion criteria were as follows:

1. age between 16 and 45 years, because of the young onset age of BD and in order to avoid the interference of estrogen level changes in postmenopausal women;
2. Chinese Han nationality;
3. first-time BD diagnosis independently performed by two experienced psychiatrists based on clinical interviews and Diagnostic and Statistical Manual of Mental Disorder-V (DSM-V) criteria;
4. disease duration of less than or equal to 5 years;
5. no current use of any psychotropic drug.

Exclusion criteria were:

1. presence of any concurrent severe medical condition, such as decompensated heart or metabolic dysfunction;
2. presence of comorbid neuropsychiatric disorders that meet International Classification of diseases-10 (ICD-10) or DSM-V diagnostic criteria, such as mental retardation, dementia, and other mental illnesses;
3. current pregnancy or lactation;
4. refusal to participate.

The control group was comprised of healthy participants who had never been diagnosed nor had a positive family history of any neuropsychiatric disorder. They were matched in age and gender to the BD group and were recruited from the Health Management Center, The Second Xiangya Hospital of Central South University, China. Other exclusion criteria for the control group were the same as those mentioned above for the patient group.

According to our previous study on BMD in individals with schizophrenia, we estimated the sample size for this study. For BMD lumbar-total, when power equals to 0.9 and alpha equals to 0.05, 33 samples for each group, 66 samples in total, were required. For BMD hip-total, when power equals to 0.9 and alpha equals to 0.05, 45 samples in each group, 90 samples in total were required. PASS 15 was used to calculate the sample size (Two-Sample T-Tests Allowing Unequal Variance) (17).

This study provided free psychological evaluation and BMD determination for all participants, without any additional subsidy, and was approved by the Ethics Committee of the Second Xiangya Hospital of Central South University, Changsha, China. All included participants voluntarily agreed to participate and signed informed consent terms.

### Sociodemographic and Clinical Assessment

The demographic and clinical information of the participants was collected during their clinic visits using questionnaires, through which information about gender, age, disease duration, family history of bone fractures, smoking and drinking history were obtained. Additional physical assessments included anthropometric measurements such as weight and height. The Hamilton Depression Scale-17 (HAMD-17), the Young Mania Rating Scale (YMRS) and the Hypomania Check List (HCL-32) were used to appraise BD-related symptoms. HAMD-17 (18) and YMRS (19) were applied by an experienced physician, while HCL-32 (20, 21), a self-rating scale, was accordingly completed by patients themselves under the supervision of a physician. All of the used instruments and scales were in its Chinese version. For HAMD-17, the patients were rated by a physician on 17 items scored on five-point scale. YMRS is an 11-item five-point multiple choice diagnostic questionnaire to measure mania. HCL-32 is a questionnaire with 32 items to identify hypomanic features to help recognize bipolar spectrum disorder.

On an empty stomach, blood samples were obtained from all the subjects between 6 AM and 9 AM. The samples were centrifuged for 10 min at 3,000 rpm by using a table-top high-speed centrifuge (TDZ5-WS, China) to obtain 2 ml of serum. Triglyceride (TG), total cholesterol (TC), low-density lipoprotein cholesterol (LDL-C), HDL-C, and fasting blood glucose (FBG) levels were measured using an automatic biochemical analyzer (7170A, Japan).

### Bone Mineral Density Assessment

In this study, the BMD of the lumbar spine (L1–L4) and the left hip, including the femoral neck (Neck), trochanter of femoral (Troch), and Ward's triangle (Ward's), were measured using dual-energy X-ray absorptiometry (Discovery Wi; S/N 87556, US) in individuals with BD and healthy controls. The categories for diagnosis are: normal (T-score −1 and above), low bone mass (T-score between −1 to −2.5), osteoporosis (T-score below −2.5). This standard provided a quantifiable indicator of osteoporosis and low bone mass (5) and was used in our binary logistic regression analysis. All BMD measurements were performed in the Second Xiangya Hospital of Central South University.

### Data Analysis

Firstly, variables were checked for normality and homogeneity of variance. We used PASS 15 to calculate the power of primary analysis and subgroup analysis, all of which had passed 90% power. The general and the BMD data of the drug-naïve BD and the healthy control groups were compared using the Student t-test (measurement data) and the chi-square test (enumeration data). Then, to analyze the data, individuals with BD were further divided into the low-weight (body mass index [BMI] < 18.5, N = 8), normal weight (18.5 ≤ BMI < 24, N = 41), and overweight (BMI ≥ 24, N = 12) groups, according to the Chinese guidelines for prevention and control of overweight and obesity (22, 23). In the BMI subgroups, the differences in BMD were compared using variance analysis. Since we were also interested in the gender differences of BMD in BD, we further analyzed the data by gender subgroup. The BMD of the gender subgroup was analyzed using the Student t-test. A binary logistic regression analysis was run with the T-score diagnose categories in each positions as the dependent variable, and the diagnosis, BMI, gender, age, and the levels of TG, TC, HDL-C, LDL-C, and FBG as independent variables. The risk of osteoporosis and low bone mass of the BD and the healthy control groups were compared, and the odds ratio (OR) derived from the binary logistic regression analysis was used to correct the relevant variables. The correlation results were Bonferroni corrected at p < 0.05. All data were analyzed using the SPSS 23 and PASS 15.0.5.

## Results

### Sociodemographic and Clinical Assessment

This study included 61 bipolar disorder participants and 95 healthy controls. We used PASS 15.0.5 to calculate the power of our study, using BMD total-hip as the main result. Our sample sizes achieved 95.29% power to reject the null hypothesis of equal means. Participants were 61 individuals with bipolar I disorder (n = 26) or bipolar II disorder (N = 35). A total of 48 patients (78.7%) entered in a bipolar I or II depressed episode, 6 patients (9.8%) in a manic or hypomanic episode, and 7 patients (11.5%) in a mixed or sub-threshold mixed episode. The drug-naive individuals with BD and the healthy controls showed no significant difference in gender composition, age, height, body weight, BMI, and smoking or drinking history. The average duration of illness was 2.46 ± 1.82 years. The HAMD-17, YMRS, and HCL-32 scores of individuals with BD were 21.15 ± 6.48, 9.00 ± 6.34, and 20.67 ± 4.42, respectively (**Table 1**).

**Table 1:** General information in BD group and healthy controls^a^.

	Bipolar disorder (BD, n = 61)	Healthy control (HC, n = 95)	Test statistics^b^	p	Relationship
Gender (M, W)	(18, 43)	(39, 56)	2.135	0.174	–
Age (y)	21.82 ± 5.36	24.46 ± 10.19	−1.866	0.064	–
Height (m)	1.63 ± 0.07	1.66 ± 0.08	−1.825	0.070	–
Weight (kg)	58.32 ± 11.47	60.39 ± 12.50	−1.041	0.299	–
BMI (kg/m^2^)	21.73 ± 3.31	21.86 ± 3.50	−0.225	0.822	–
Smoking (Y, N)	(13, 47)	(17, 78)	0.335	0.563	–
Drinking (Y, N)	(7, 53)	(4, 91)	3.101	0.078	–
Disease Duration(y)	2.46 ± 1.82	–	–	–	–
HAMD-17	21.15 ± 6.84	–	–	–	–
YMRS	9.00 ± 6.34	–	–	–	–
HCL-32	20.67 ± 4.42	9.69 ± 4.115	10.881	<0.001	BD > HC
TG (mmol/L)	1.08 ± 0.71	1.20 ± 1.13	−0.687	0.493	–
TC (mmol/L)	3.88 ± 0.82	4.13 ± 0.68	−1.905	0.059	–
HDL-C (mmol/L)	1.20 ± 0.28	1.33 ± 0.29	−2.665	0.009	BD < HC
LDL-C (mmol/L)	2.32 ± 0.70	2.30 ± 0.56	0.195	0.846	–
FBG (mmol/L)	4.22 ± 1.08	4.54 ± 0.48	−2.111	0.038	BD < HC

BMI, body mass index, which is calculated as weight in kilograms divided by height in meters squared; TG, triglycerides; TC, total cholesterol; HDL-C, high-density lipoprotein cholesterol; LDL-C, low-density lipoprotein cholesterol; FBG, fasting blood glucose.

a Data are presented as mean ± SD.

b Test statistics: χ2 Test for categorical variables and Student t-test for continuous variables.

### Laboratory Results

The laboratory results of the individuals with drug-naive BD and the healthy controls are presented in **Table 1**. No significant difference was observed between the two groups in terms of TG, TC, and LDL-C levels. The drug-naive BD group had significantly lower HDL-C levels (t = −2.665, p = 0.009 < 0.05) and FBG levels (t = −2.111, p = 0.038 < 0.05) than the healthy control group.

### Bone Mineral Density

As shown in **Table 2**, the BMD of the drug-naive BD group was significantly lower in L1 (t = −3.400, p = 0.001 < 0.05), L3 (t = −2.008, p = 0.046 < 0.05), Neck (t = −4.584, p < 0.001 < 0.05), Troch (t = −4.291, p < 0.001 < 0.05), Ward's (t = −3.529, p = 0.001 < 0.05), and total hip (t = −2.721, p = 0.007 < 0.05) than the healthy control group. No significant differences of BMD was observed in other measured regions.

**Table 2:** BMD value in BD group and healthy controls^a^.

BMD (g/cm^2^)	Bipolar disorder (BD, n = 61)	Health control (HC, n = 95)	Test statistics^b^	p	Relationship
L1	0.89 ± 0.10	0.95 ± 0.11	−3.400	0.001	BD < HC
L2	1.16 ± 1.50	1.01 ± 0.12	0.974	0.332	–
L3	1.00 ± 0.12	1.03 ± 0.11	−2.008	0.046	BD < HC
L4	0.98 ± 0.10	1.01 ± 0.11	−1.581	0.116	–
Total-lumbar	0.96 ± 0.10	0.99 ± 0.14	−1.550	0.123	–
Neck	0.80 ± 0.10	0.89 ± 0.13	−4.584	<0.001	BD < HC
Troch	0.66 ± 0.08	0.72 ± 0.10	−4.291	<0.001	BD < HC
Ward's	0.76 ± 0.13	0.84 ± 0.15	−3.529	0.001	BD < HC
Total-hip	0.90 ± 0.11	0.96 ± 0.13	−2.721	0.007	BD < HC

L1, BMD of L1; L2, BMD of L2; L3, BMD of L3; L4, BMD of L4; Total-lumbar, BMD of Total-lumbar; Neck, femoral neck; Troch, trochanter of femoral; Ward's, Ward's triangle

a Data are presented as mean ± SD.

b Test statistics: Student t-test for continuous variables.

### Subgroup Analysis Based on BMI

We used PASS 15 to calculate the power of our study, using BMD total-hip as the main outcome. In a one-way ANOVA study, sample sizes of 8, 41, and 12 are obtained from the three groups whose means are to be compared. A total sample of 61 subjects achieves 93% power to detect differences between means *versus* the alternative of equal means using an F test with a 0.0500 significance level.

The subgroups defined by BMI showed no significant difference in gender composition, age, height, smoking, drinking, duration of disease, HAMD-17, YMRS, and HCL-32. The participants showed no significant difference in HDL-C and FBG levels. The overweight group had significantly higher TG (F = 16.273, p < 0.001 < 0.05), TC (F = 5.516, p = 0.007 < 0.05), and LDL-C (F = 7.874, p = 0.001 < 0.05) levels than the other two groups (**Table 3**).

**Table 3:** General information and BMD values in BMI defined subgroups of BD patients^a^.

	Low-weight (LW, n = 8)	Normal weight (NW, n = 41)	Overweight (OW, n = 12)	F^b^	p	LW vs NW	LW vs OW	NW vs OW	Relationship
Weight (kg)	47.18 ± 3.31	55.11 ± 5.49	76.73 ± 10.12	66.003	<0.001	0.002	< 0.001	< 0.001	O > N > L
TG (mmol/L)	0.83 ± 030	0.88 ± 0.49	1.98 ± 0.92	16.273	<0.001	0.852	< 0.001	< 0.001	O > N, L
TC (mmol/L)	3.75 ± 0.88	3.72 ± 0.77	4.57 ± 0.67	5.516	0.007	0.912	0.040	0.002	O > N, L
LDL-C (mmol/L)	2.16 ± 0.61	2.16 ± 0.60	3.00 ± 0.74	7.874	0.001	0.972	0.011	< 0.001	O > N, L
L1 (g/cm2)	0.83 ± 0.08	0.90 ± 0.10	0.94 ± 0.11	2.716	0.075	0.096	0.023	0.216	–
L2 (g/cm2)	0.90 ± 0.10	1.25 ± 1.83	1.01 ± 0.11	0.250	0.780	0.547	0.865	0.637	–
L3 (g/cm2)	0.91 ± 0.08	1.00 ± 0.11	1.05 ± 0.13	3.401	0.040	0.051	0.012	0.210	O > L
L4 (g/cm2)	0.91 ± 0.10	0.98 ± 0.09	1.03 ± 0.12	2.993	0.058	0.098	0.018	0.160	–
Total- lumbar (g/cm2)	0.89 ± 0.09	0.96 ± 0.09	1.01 ± 0.11	3.642	0.032	0.065	0.009	0.129	O > L
Neck (g/cm2)	0.73 ± 0.06	0.79 ± 0.10	0.87 ± 0.10	5.900	0.005	0.171	0.002	0.007	O > N, L
Troch (g/cm2)	0.60 ± 0.06	0.65 ± 0.07	0.72 ± 0.08	7.098	0.002	0.104	0.001	0.004	O > N, L
Ward's (g/cm2)	0.75 ± 0.09	0.74 ± 0.12	0.83 ± 0.16	2.246	0.115	0.926	0.158	0.041	–
Total-hip (g/cm2)	0.87 ± 0.11	0.88 ± 0.09	0.99 ± 0.12	5.700	0.005	0.801	0.013	0.005	O > N, L

TG, triglyceride; TC, total cholesterol; LDL-C, low-density lipoprotein cholesterol; L1, bone mineral density (BMD) of L1; L2, BMD of L2; L3, BMD of L3; L4, BMD of L4; Total-lumbar, BMD of Total-lumbar; Neck, femoral neck; Troch, trochanter of femoral; Ward's, Ward's triangle

a Data are presented as mean ± SD.

b P-value for the omnibus analysis testing for overall differences between the three groups on the continuous variables is based on ANOVA. When the overall omnibus analysis P-value was significant, the pair-wise comparisons were performed.

In the BD group, the BMD of the low-weight, the normal weight and the overweight groups were different in L3 (F = 3.401, p = 0.040 < 0.05), total lumbar (F = 3.642, p = 0.032 < 0.05), Neck (F = 5.900, p = 0.005 < 0.05), Troch (F = 7.098, p = 0.002 < 0.05), and total hip (F = 5.700, p = 0.005 < 0.05). No significant differences of BMD were found in other regions (**Table 3**). Similar results took place for the control group (**Supplementary Table 1**).

### Subgroup Analysis Based on Gender

The gender subgroup analysis of BD patients showed no significant differences in age, drinking, duration of disease, HAMD, YMRS, and HCL-32, while the height, weight, BMI, and smoking rate of the male group were higher than the female group. In laboratory results, the participants showed no significant differences in TC, HDL-C, and FBG levels between gender. The male group had significantly higher TG (t = 2.484, p = 0.021 < 0.05) and LDL-C (t = 2.105, p = 0.046 < 0.05) levels than the female group (**Supplementary Table 2**). The gender subgroup analysis in healthy controls showed no significant differences in age, smoking and drinking history. Height, weight, and BMI were lower in the female in comparison to the male group (**Supplementary Table 3**).

By analyzing the data in gender subgroups, both of the BD group and the healthy control group had higher BMD in males. The results illustrated that only the Neck was statistically different between gender in the BD group (t = 2.115, p = 0.039), whereas the measurements of healthy controls showed differences in Neck (t = 5.352, p < 0.001), Troch (t = 3.799, p < 0.001 = 0.000), Ward's (t = 3.991, p < 0.001), and total hip (t = 2.055, p = 0.043). The difference in BMD in the other regions between the male and female subgroups was not found.

### Logistic Regression

The variance analysis of the regression model showed that the regression equation of L1, Neck, Troch, and total hip had statistical significance (p < 0.05). The binary logistic regression analysis showed that the low BMD in multiple regions was associated with BD diagnosis and BMI (**Table 4**, **Supplementary Table 4**). Moreover, being female and older were risk factors for low bone mass in L1. Additionally, BD diagnosis was a risk factor for low BMD in L1 (OR = 3.891, 95% confidence interval [CI] = 1.522 to 9.948), Neck (OR = 4.715, 95% CI = 1.581 to 14.059), Troch (OR = 7.039, 95% CI = 1.820 to 27.217), and total hip (OR = 6.176, 95% CI = 1.554 to 24.551) (**Supplementary Table 5**).

**Table 4:** The binary logistic regression analysis^a^.

Regions	Model		Diagnose		BMI		Gender		Age	
	χ^2^	p	β	p	β	p	β	p	β	p
L1	27.562	0.001	1.359	0.005	0.191	0.028	1.761	<0.001	−0.018	0.005
Neck	20.945	0.013	1.551	0.005	0.248	0.044	−0.084	0.885	0.004	0.952
Troch	21.384	0.011	1.951	0.005	0.376	0.018	−0.382	0.602	−0.012	0.846
Total-hip	19.138	0.024	1.821	0.010	0.385	0.024	1.129	0.110	−0.066	0.319

a The binary logistic regression analysis was run with diagnose of low BMD, normal BMD (T-score of BMD > −1) or not, as the dependent variable and the diagnosis of BD, BMI, age, gender, and the levels of TG, TC, HDL-C, LDL-C, and FBG were the independent variables.

## Discussion

Prior research indicates that bipolar disorder is associated with decreased BMD and osteoporosis (24). However, results are conflicting since samples are often subject to concurrent use of psychotropic medications, some of which are themselves independent risk factors for low BMD (14, 25). Furthermore, hardly any study objectively assessed BMD in drug-naïve patients with BD. To the best of our knowledge, this is the first study to objectively assess BMD in a sample of newly diagnosed drug-naïve patients with BD belonging to the Chinese Han nationality.

Our results show that patients with BD had low BMD in multiple sites in comparison to age- and gender-matched healthy controls, a finding that was further confirmed in binary logistic regression analysis. Comprehensively, our findings suggest that BD itself may be an independent risk factor for decreased BMD. Our findings are consistent with the results of a large retrospective cohort study in Taiwan, which included 47,271 patients with BD and 189,084 matching controls. In that study, Hsu and coworkers showed that the risk of fracture in patients with BD (17.6%) was significantly higher than that in the control group (11.7%) (P < 0.001), and the hazard ratio (HR) was 1.33 (95% CI = 1.23 to 1.48, P < 0.001). Furthermore, the COX regression model analysis illustrated that BD itself was an independent risk factor of osteoporosis (10). A systematic review of 344,497 participants showed an increased risk of fracture in patients with BD (range = 20–80%), and the fracture-free survival time of those with BD decreased substantially with advancing age and in female (10–30% shorter than men). Fracture incidences in individuals with and without BD were 21.4 and 10.8 per 1,000 person-year, respectively (24).

Research on BD has focused on the bone loss in pre- or post-menopausal women with long-term treatment (11, 26). However, our study found that this risk of bone metabolism exists not only in long-term treatment women but also in men and young drug-naïve people with BD. Although prior studies suggested that long-term drug therapy may be the main risk factor for bone mass reduction in BD, our study shows that decreased BMD is already present in this population even before psychotropic treatment was started.

To our knowledge, no study has analyzed the risk factors for low BMD in drug-naïve individuals with BD yet. In our study, the binary logistic regression analysis showed that factors, including BD itself, gender, age, and BMI, can influence bone density, which indicated that suffering from BD, being female, having low BMI are risk factors for low bone mass and osteoporosis.

The association between BD and low BMD could be explained by shared biological pathways that are common both to bone metabolism and BD neurobiology. Those could include immune (16), neuroendocrine (27, 28), and mitochondrial dysfunction (29, 30), in addition to excessive oxidative stress (15).

Some evidence revealed that the pathogenesis of BD is associated with immune disorders affecting the central and peripheral nervous systems (31). As an important cytokine in immune regulation, TNF- α takes part in the pathogenesis of BD (32) and influences bone absorption by inducing the production of the RANKL protein and promoting osteoclastogenesis (33). Therefore, TNF- α inflammation-induced osteoclastogenesis may be more active in patients with BD, increasing the risk of fracture in these patients. Additionally, other proinflammatory cytokines/mediators that have been implicated in the pathogenesis of BD, including CRP (34) and IL-6 (35), also increase osteoclast activity and bone resorption, thereby reducing BMD (36).

BD has also been linked to disorders of the hypothalamic-pituitary-adrenal axis (HPA) (37) and hypothalamic-pituitary-thyroid axis (HPT) (38). HPA axis dysfunction and associated glucocorticoid resistance have been shown across different phases of BD (27). Increased HPA activity can lead to the suppression of growth hormone-releasing hormone (GHRH) and gonadotropin-releasing hormone (GnRH), thereby decreasing the release of estrogen, testosterone, and growth hormone (GH), which are important regulators of bone metabolism (39). Barbuti et al. reported that thyroid autoimmunity may be an independent risk factor for bipolar disorder (40). Meanwhile, Lithium coud significantly increase the TSH and decrease fT4 level in bipolar patients (41). Furthermore, it has been reported that TSH could activate osteoblasts and inhibit osteoclast activity, resulting in osteoprotection (42) while the absence of TSH signalling contributes to bone loss (28). Adding to the effects of biological factors, behavioral patterns such as decreased activity level, drug abuse (43), and overeating (44) could increase the risk of low BMD and osteoporosis in individuals with BD (45).

In order to investigate the association between BMI with BMD, we further divided the patient group into three subgroups according to their BMI. In this subgroup analysis, we found that individuals with lower BMI had also significantly lower BMD, which is in agreement with previous studies, albeit in distinct populations. A meta-analysis of nearly 400,000 women showed that most osteoporotic (81%) and hip (87%) fractures occurred in non-obese individuals (46), with similar results being also observed in patients with schizophrenia (47). Protective factors may be related to insulin and insulin amyloid secreted by pancreatic beta cells. The former is able to directly stimulate osteoblasts, promoting the proliferation of these cells. Insulin amyloid, in addition to induce osteoblast spread, also can inhibit bone absorption, with the consequent increase in bone mass and density (48). Nonetheless, different results were observed in a sample of patients with schizophrenia, in a meta-analysis showing that higher BMI was associated with decreased BMD in patients with schizophrenia (49). Consequently, the nature of the relation between BMI and BMD remains elusive.

Based on gender subgroup analysis, we found that being female was a risk factor for low BMD both drug-naïve patients with BD and healthy controls. While this relationship went in the same direction for both groups, it was more pronounced in healthy participants. Overall, this is consistent with a survey that disclosed that females presented a trend towards lower BMD and higher fracture risk in comparison to males, especially after menopausal decrease of estrogen levels (50). However, we found that gender-related differences in BMD in patients with BD was only observed in one site and was less evident than in normal controls. This finding may be due to our relatively small sample size, which may have left our analysis of subgroups underpowered. Additionally, behavioral aspects may also have played a role. For instance, leveling of gender-related BMD might result from a higher smoking rate in male individuals with BD, since smoking was found to be directly related to decreased bone density (51). Also, a meta-analysis indicated that a higher percentage of males was associated with a higher probability of suffering from osteopenia and osteoporosis (49). Moreover, while some studies suggested that although postmenopausal females have a higher risk of fracture, elderly males tend to have poorer outcomes and treatment rates after fracture. Therefore, regardless of gender, the treatment rate of osteoporosis in high-risk people needs to be improved (52). Gender differences should be considered in future studies involving patients with BD.

As for the limitations of our study, we did not assess variables that could potentially confound analysis, such as individual levels of physical activity, diet, drug abuse and vitamin D levels Additionally, our findings cannot be generalized to the BD population overall since our sample included only newly diagnosed drug-naïve patients belonging to the Chinese Han nationality. Moreover, as this study had a cross-sectional design, we could only demonstrate an association between BD and comorbid low BMD in drug-naïve patients, without establishing a causal relationship. Future well-designed prospective studies with large samples should further longitudinally assess BMD in BD samples, ideally also including severe related outcomes such as bone fractures, need for surgical interventions, and permanent physical disability. Markers of bone turnover other than BMD, which were not included in our study, should also be incorporated in comprehensive evaluations of bone metabolic disorders in patients with BD. Finally, although the sample size of this study has reached the desired statistical power, it is still a small sample study, especially when considering subgroup analysis.

## Conclusion

BMD in multiple sites of drug-naive individuals with BD was significantly lower than that of the healthy controls in a sample of the Chinese Han nationality. Risk factors for reduced BMD were BD diagnosis, female gender, and lower BMI. Our findings are consistent with the hypothesis that BD and reduced BMD/osteoporosis share common pathological mechanisms. Bone density reduction or osteoporosis is attached to increased fracture risk in patients with BD, which may lead to severe outcomes such as permanent disability and increased mortality. Therefore, studies focusing on prevention and intervention strategies should be considered, including changing lifestyle, improving diet and exercise, promoting physical activity, preventing falls, and providing patients with biopsychosocial medical treatment. In conclusion, our preliminary study suggests that individuals with BD suffer from low BMD or osteoporosis even before starting medication treatment and low BMI and female gender are risk factors for these adverse outcomes.

## Data Availability Statement

The datasets generated for this study are available on request to the corresponding authors.

## Ethics Statement

This study was approved by the Ethics Committee of the Second Xiangya Hospital of Central South University. Written informed consent was obtained from the participants for their participation in this study.

## Author Contributions

HW, BW, and JC conducted and designed the study. SL, YQ, ZT, YY, CW, YT, and LW collected the data. SL and HW analyzed and interpreted the data. SL drafted the manuscript. BW, DK, TH, and XH provided critical text revision. All authors have contributed to and approve the final manuscript.

## Funding

This work was supported by the National Natural Science Foundation of China (Grant number 81501163) and National Natural Science Foundation of China (Grant number 81971258).

## Conflict of Interest

The authors declare that the research was conducted in the absence of any commercial or financial relationships that could be construed as a potential conflict of interest.
