# Supplementary material for: Association Between Bipolar Disorder and Low Bone Mass: A Cross-Sectional Study With Newly Diagnosed, Drug-Naïve Patients
Source: Front Psychiatry. 2020 Jun 10;11:530. doi: 10.3389/fpsyt.2020.00530 (PMC7299052; doi:10.3389/fpsyt.2020.00530)
Supplement: Supplementary file 1 [file DataSheet_1.docx]

Supplementary Table 1 The general information and BMD value in subgroups of BMI in healthy control group ^a^.

|  | low-weight  (LW, n=14) | normal weight  (NW, n=61) | overweight  (OW, n=20) | F^b^ | p | | LW vs NW | | LW vs OW | | NW vs OW | | Relationship |
| --- | --- | --- | --- | --- | --- | --- | --- | --- | --- | --- | --- | --- | --- |
| Weight  (kg) | 48.17±4.70 | 57.26±7.57 | 78.50±9.49 | 78.346 | ＜0.001 | ＜0.001 | | ＜0.001 | | ＜0.001 | | O＞N＞L | |
| TG  (mmol/L) | 0.80±0.23 | 0.98±0.86 | 2.10±1.64 | 9.535 | ＜0.001 | 0.574 | | 0.001 | | ＜0.001 | | O＞N, L | |
| HDL-C  (mmol/L) | 1.57±0.23 | 1.36±0.27 | 1.10±0.20 | 13.837 | ＜0.001 | 0.011 | | ＜0.001 | | ＜0.001 | | O＜N＜L | |
| L1  (g/cm2) | 0.89±0.06 | 0.96±0.11 | 0.98±0.10 | 3.185 | 0.046 | 0.038 | | 0.016 | | 0.369 | | L＜N, O | |
| L2  (g/cm2) | 0.94±0.07 | 1.01±0.12 | 1.03±0.13 | 3.018 | 0.054 | 0.032 | | 0.022 | | 0.522 | | - | |
| L3  (g/cm2) | 0.98±0.08 | 1.04±0.11 | 1.05±0.11 | 1.897 | 0.156 | 0.073 | | 0.080 | | 0.760 | | - | |
| L4  (g/cm2) | 0.98±0.07 | 1.01±0.12 | 1.02±0.10 | 0.686 | 0.506 | 0.348 | | 0.251 | | 0.634 | | - | |
| Total-  lumbar  (g/cm2) | 0.95±0.07 | 0.99±0.16 | 1.02±0.10 | 1.082 | 0.343 | 0.314 | | 0.145 | | 0.412 | | - | |
| Neck  (g/cm2) | 0.82±0.12 | 0.87±0.12 | 1.00±0.12 | 11.743 | ＜0.001 | 0.183 | | ＜0.001 | | ＜0.001 | | O＞N, L | |
| Troch  (g/cm2) | 0.67±0.08 | 0.71±0.09 | 0.80±0.11 | 10.460 | ＜0.001 | 0.193 | | ＜0.001 | | ＜0.001 | | O＞N, L | |
| Ward’s  (g/cm2) | 0.82±0.15 | 0.82±0.15 | 0.92±0.13 | 3.415 | 0.037 | 0.865 | | 0.048 | | 0.014 | | O＞N, L | |
| Total-hip  (g/cm2) | 0.88±0.12 | 0.94±0.11 | 1.07±0.14 | 14.297 | ＜0.001 | 0.081 | | ＜0.001 | | ＜0.001 | | O＞N, L | |

TG, triglyceride; TC, total cholesterol; LDL-C, low-density lipoprotein cholesterol; L1, bone mineral density (BMD) of L1; L2, BMD of L2; L3, BMD of L3; L4, BMD of L4; Total-lumbar, BMD of Total-lumbar; Neck, femoral neck; Troch, trochanter of femoral; Ward’s, Ward’s triangle

^a^ Data are presented as mean ± SD.

^b^ P-value for the omnibus analysis testing for overall differences between the three groups on the continuous variables is based on ANOVA. When the overall omnibus analysis P-value was significant, the pair-wise comparisons were performed.

Supplementary Table 2 Subgroup of gender in BD

|  | Male(n=18) | Female (n=43) | t,  χ2 | p | Relationship |
| --- | --- | --- | --- | --- | --- |
| Age (y) | 21.28±4.76 | 22.05±5.64 | -0.507 | 0.614 | - |
| Height (m) | 1.71±0.07 | 1.60±0.05 | 6.578 | ＜0.001 | M＞F |
| Weight (kg) | 68.08±12.92 | 54.23±7.89 | 4.229 | ＜0.001 | M＞F |
| BMI (kg/m2) | 23.21±3.49 | 21.12±3.07 | 2.331 | 0.023 | M＞F |
| Smoking (Y, N) | (8,10) | (5,37) | 7.861 | 0.005 | M＞F |
| Drinking (Y, N) | (4,14) | (3,39) | 2.780 | 0.095 | - |
| HAMD-17 | 19.78±8.06 | 21.74±6.27 | -1.017 | 0.313 | - |
| YMRS | 11.50±7.60 | 7.93±5.48 | 1.803 | 0.084 | - |
| TG (mmol/L) | 1.49±0.95 | 0.90±0.49 | 2.484 | 0.021 | M＞F |
| TC (mmol/L) | 4.18±0.97 | 3.75±0.72 | 1.718 | 0.098 | - |
| HDL-C (mmol/L) | 1.14±0.20 | 1.24±0.31 | -1.497 | 0.141 | - |
| LDL-C (mmol/L) | 2.65±0.89 | 2.17±0.55 | 2.105 | 0.046 | M＞F |
| FBG (mmol/L) | 4.57±1.69 | 4.07±0.62 | 1.227 | 0.235 | - |
| L1 (g/cm2) | 0.91±0.08 | 0.89±0.11 | 0.956 | 0.343 | - |
| L2 (g/cm2) | 0.99±0.07 | 1.23±1.79 | -0.564 | 0.575 | - |
| L3 (g/cm2) | 1.03±0.11 | 0.98±0.12 | 1.348 | 0.183 | - |
| L4 (g/cm2) | 0.99±0.08 | 0.97±0.11 | 0.558 | 0.579 | - |
| Total-lumbar(g/cm2) | 0.98±0.07 | 0.95±0.11 | 0.986 | 0.328 | - |
| Neck (g/cm2) | 0.84±0.09 | 0.78±0.11 | 2.115 | 0.039 | M＞F |
| Troch (g/cm2) | 0.67±0.06 | 0.65±0.09 | 0.948 | 0.347 | - |
| Total-hip(g/cm2) | 0.93±0.09 | 0.89±0.11 | 1.258 | 0.213 | - |
| Ward’s (g/cm2) | 0.76±0.13 | 0.76±0.13 | 0.122 | 0.904 | - |

Supplementary Table 3 Subgroup of gender in health control

|  | Male(n=38) | Female (n=48) | t,  χ2 | p | Relationship |
| --- | --- | --- | --- | --- | --- |
| Age (y) | 23.64±2.61 | 25.04±13.11 | -0.654 | 0.514 | - |
| Height (m) | 1.72±0.04 | 1.61±0.06 | 9.355 | ＜0.001 | M＞F |
| Weight (kg) | 69.67±12.59 | 54.07±7.65 | 6.813 | ＜0.001 | M＞F |
| BMI (kg/m2) | 23.50±4.07 | 20.72±2.51 | 3.786 | ＜0.001 | M＞F |
| Smoking (Y, N) | (10,29) | (7,49) | 2.702 | 0.100 | - |
| Drinking (Y, N) | (3,36) | (1,55) | 1.898 | 0.158 | - |
| TG (mmol/L) | 1.66±1.55 | 0.83±0.34 | 3.259 | 0.002 | M＞F |
| TC (mmol/L) | 4.21±0.75 | 4.07±0.61 | 0.996 | 0.322 | - |
| HDL-C (mmol/L) | 1.20±0.24 | 1.44±0.28 | -4.021 | ＜0.001 | M＜F |
| LDL-C (mmol/L) | 2.38±0.64 | 2.24±0.48 | 1.045 | 0.300 | - |
| FBG (mmol/L) | 4.52±0.53 | 4.56±0.44 | -0.338 | 0.736 | - |
| L1 (g/cm2) | 0.97±0.11 | 0.94±0.11 | 0.945 | 0.347 | - |
| L2 (g/cm2) | 1.02±0.12 | 1.00±0.12 | 1.046 | 0.298 | - |
| L3 (g/cm2) | 1.05±0.11 | 1.00±0.11 | 1.054 | 0.295 | - |
| L4 (g/cm2) | 1.02±0.10 | 1.02±0.11 | 0.649 | 0.518 | - |
| Total-lumbar(g/cm2) | 0.99±0.18 | 0.99±0.10 | -0.044 | 0.965 | - |
| Neck (g/cm2) | 0.96±0.12 | 0.83±0.11 | 5.352 | ＜0.001 | M＞F |
| Troch (g/cm2) | 0.77±0.10 | 0.69±0.09 | 3.799 | ＜0.001 | M＞F |
| Total-hip(g/cm2) | 1.02±0.12 | 0.92±0.12 | 3.991 | ＜0.001 | M＞F |
| Ward’s (g/cm2) | 0.88±0.14 | 0.82±0.15 | 2.055 | 0.043 | M＞F |

Supplementary Table 4 The binary logistic regression analysis ^a^

| regions | TG | | TC | | HDL-C | | LDL-C | | FBG | |
| --- | --- | --- | --- | --- | --- | --- | --- | --- | --- | --- |
|  | β | p | β | p | β | p | β | p | β | p |
| L1 | -0.009 | 0.987 | 0.022 | 0.986 | -0.545 | 0.724 | 0.220 | 0.854 | -0.208 | 0.472 |
| Neck | 0.453 | 0.573 | -0.277 | 0.879 | -0.662 | 0.753 | -0.432 | 0.823 | 0.331 | 0.323 |
| Troch | -0.234 | 0.786 | -0.007 | 0.997 | 0.212 | 0.929 | -0.406 | 0.846 | -0.269 | 0.446 |
| Total-hip | -0.969 | 0.283 | 1.489 | 0.462 | -3.560 | 0.147 | 0.967 | 0.682 | -0.320 | 0.389 |

^a^The binary logistic regression analysis was run with normal BMD (T-score of BMD＞-1) or not as the dependent variable, and the diagnosis of BD, BMI, age, gender and the levels of TG, TC, HDL-C, LDL-C and FBG were the independent variables.

Supplementary Table 5 The risk of osteoporosis and low bone mass of the BD and the healthy control groups

|  | OR | 95% CI | |
| --- | --- | --- | --- |
|  |  | Lower | Upper |
| L1 | 3.891 | 1.522 | 9.948 |
| L2 | 2.534 | 0.733 | 8.756 |
| L3 | 1.660 | 0.562 | 4.899 |
| L4 | 1.574 | 0.684 | 3.621 |
| Total-lumbar | 1.363 | 0.397 | 4.678 |
| Neck | 4.715 | 1.581 | 14.059 |
| Troch | 7.039 | 1.820 | 27.217 |
| Total-hip | 6.176 | 1.554 | 24.551 |
| Ward’s | 5.054 | 1.497 | 17.059 |
